# Juvenile xanthogranuloma: a case report and review of the literature

**DOI:** 10.1186/1756-0500-7-174

**Published:** 2014-03-26

**Authors:** Laura Pajaziti, Syzana Rexhepi Hapçiu, Artina Pajaziti

**Affiliations:** 1 Clinic of Dermatovenerology, University of Prishtina, Rr. “Spitalit” p.n., 10000 Prishtina, Kosovo; 2 University of Prishtina, Faculty of Medicine, 10000 Prishtina, Kosovo

**Keywords:** Juvenile xanthogranuloma, Nodule

## Abstract

**Background:**

Juvenile xanthogranuloma is a rare disorder which may be present at birth, or appears in infancy. It can also occur in adults of all ages; appears with lesions that may be solitary or multiple nodules several millimeters in diameter. The predilection sites are head and neck, but it may occur on the extremities and trunk also. There can also be involved internal organs such as lung, kidney, gastrointestinal tract, etc. The most frequent extracutaneous location is the eye.

**Case presentation:**

We report a case of juvenile xanthogranuloma in a male child with onset in the fourth month of life. He presented with a nodule 8 millimeters in diameter, tan-orange in color, ulcerated in the centre, located on the left corner of the left eye. A biopsy without total excision was performed. After the biopsy, the nodule enlarged to 1.5 cm in diameter and became haemorrhagic. The histologic evaluation and immunohistochemistry analysis resulted in the diagnosis of juvenile xanthogranuloma. For aesthetic reasons the nodule was removed by surgical resection.

**Conclusion:**

Juvenile xanthogranuloma is on a spectrum of histiocytic disorders, which is necessary to differentiate from maligniances in childhood by biopsy.

## Background

Juvenile xanthogranuloma (JXG) is a rare disorder, which belongs to the broad group of non - Langerhans cell histiocytosis [1]. JXG may be present at birth, but it mostly arises during the first year of life [2]. It is characterized by one or more nodules with the predilection sites on the head and neck, although the appearance on the trunk, extremities and extracutaneous locations has been reported also [3,4]. Skin lesions are self- limited and vary in size. They are reddish or yellowish benign papules or nodules, which do not require treatment [4,5]. The eye, particularly the uveal tract, is the most frequent site of extracutaneous involvement [6].

Histologically, JXG is composed of collections of histiocytes, foamy cells and Touton giant cells. The diagnosis of JXG is mainly clinical, but sometimes a biopsy analysis is required [7].

Skin lesions generally follow a benign course with spontaneous resolution. Extracutaneous lesions are rare and can be associated with morbidity.

## Case presentation

A five-month-old male presented with a nodule in the left corner of the left eye. He was born at 40 weeks’ gestational age, without any abnormality or history of trauma at birth. The lesion was first noted at four months of age. The nodule was 8 millimeters in diameter, tan-orange in color, with central ulceration.

**Figure 1:**
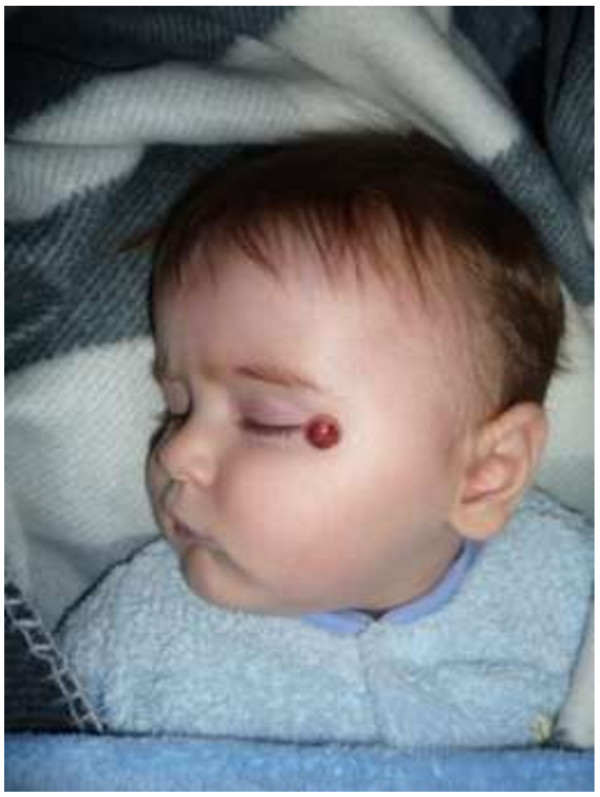
The enlarged nodule after partial biopsy.

His parents consulted a maxillofacial surgeon, who took a partial biopsy from the nodule. After the biopsy, the nodule enlarged and became hemorrhagic and the parents brought their child to the Clinic of Dermatology (Figure 1). In mean time the histological evaluation revealed ulcerated cutaneous cellular proliferation, diffuse infiltrates of mononucleated cells, some of which are characterized by their reniform nuclei, foamy histiocytes and numerous Touton giant cells, admixed with eosinophils.

Immunohistochemistry yielded results immunoreactive for CD68, weak focal positivity for S-100 protein, but negative for Langerin. These results confirmed that the histiocytes are non-Langerhans cells.

After the diagnosis was made, the child was treated by an ophthalmologist, and examination was within normal limits.

## Discussion

JXG is a benign cutaneous fibrohistiocytic lesion and a type of granulomatous process [3]. Even though the etiology is unknown, it is believed to result from disordered macrophage response to the nonspecific injury [6]. The main clinical feature is papulonodular lesion, tan – orange in color and several millimeters in diameter, which may be single or multiple. The predilection sites of occurence are the skin of head and neck, but these papulonodular lesions may occur on the trunk and extremities also. The eye is the most frequent extracutaneous location of JXG. Ocular involvement may occur without concomitant skin involvement. 0.5% of patients with cutaneous JXG are reported with ocular involvement [6,8], although in some studies it is reported a higher incidence [6,9]. Children with multiple skin lesions and those who are younger than 2 years are at greater risk for ocular involvement [3,6]. Other extracutaneous locations of JXG are the lung, heart, gastrointestinal system, central nervous system, adrenal gland, pituitary gland, bones, bone marrow, and kidney.

JXG was reported by H.G. Adamson in 1905 [10] and named in 1954 describing the appearance of cells under microscope [11]. In about 10% of cases may be present at birth, but mainly affects infants and small children. According to Zimmerman 64% of cutaneous lesions are by age 7 months and 85% before 1 year [6]. The appearance of multiple lesions is more frequent in children younger than 6 months [3], which is not our case. JXG may occur in adults of all ages too, but this onset is infrequent and tend to be more complicated [12].

The male to female ratio of cutaneous JXG is about 1.4:1 in children, while in adults no sex predilection exists [12].

Since the disease is often clinically diagnosed and not always confirmed histologically, sometimes is misdiagnosed. On the other hand, spontaneous regression of the cutaneous lesions is frequent. Therefore, the incidence of the diseased with JXG is unknown. In a survey of 122 dermatologists with an average of 12 years of practice, less than two cases per dermatologist per year were reported [12].

The diagnosis of JXG is mainly based on characteristic clinical features. The clinical differential diagnosis includes spitz nevi, mastocytomas and dermatofibromas [1].

The confirmation of clinical diagnosis can be made by skin biopsy.

Characteristic histologic findings in JXG are: dense dermal histiocytic infiltrate and Touton Giant cells which are multinucleated, with homogeneous eosinophilic cytoplasmic center and xanthomatization in the periphery [7].

Immunohistochemistry has an important role in the differential diagnosis between Langerhans cell histiocytosis (LCH) and JXG [3]. JXG lesions usually label strongly with markers CD68, factor XIIIa and often anti CD4 [13,14]. S-100 protein immunoreactivity, which is a marker for the diagnosis of LCH, is typically absent [13,14]. In most cases with JXG, S-100 protein was non reactive, but scattered cells may show weak cytoplasmic reactivity, unlike the more diffuse and intense reaction of Langerhans cells [15]. According to Dehner, neither factor XIIIa negativity, nor S-100 positivity should preclude the diagnosis of JXG [1].

## Conclusions

JXG is self-limited disorder and skin lesions usually resolve spontaneously. Treatment is required on those with extracutaneous involvement, who may have increased morbidity. Sometimes the excision is performed for diagnosis and aesthetic reasons. In our case, the nodule enlarged and became haemorrhagic after partial biopsy. In order for proper diagnosis and for aesthetic reasons the removal of nodule must be complete.

## Consent

Written informed consent was obtained from the patient’s parents for publication of this Case Report and any accompanying images. A copy of the written consent is available for review by the Editor-in-Chief of this journal.

## Abbreviations

JXG: Juvenile xanthogranuloma; LCH: Langerhans cell histiocytosis.

## Competing interests

The authors declare that they have no competing interests.

## Authors’ contributions

LP, SR and AP analyzed and interpreted the patients’ data. LP was a major contributor in writing the manuscript. All authors read and approved the final manuscript.
